# Neuraxial Cytokines in Pain States

**DOI:** 10.3389/fimmu.2019.03061

**Published:** 2020-01-28

**Authors:** Gilson Gonçalves dos Santos, Lauriane Delay, Tony L. Yaksh, Maripat Corr

**Affiliations:** ^1^Department of Anesthesiology, University of California, San Diego, La Jolla, CA, United States; ^2^Division of Rheumatology, Allergy and Immunology, University of California, San Diego, La Jolla, CA, United States

**Keywords:** cytokine, chemokine, pain, neuroimmune crosstalk, neuraxis

## Abstract

A high-intensity potentially tissue-injuring stimulus generates a homotopic response to escape the stimulus and is associated with an affective phenotype considered to represent pain. In the face of tissue or nerve injury, the afferent encoding systems display robust changes in the input–output function, leading to an ongoing sensation reported as painful and sensitization of the nociceptors such that an enhanced pain state is reported for a given somatic or visceral stimulus. Our understanding of the mechanisms underlying this non-linear processing of nociceptive stimuli has led to our appreciation of the role played by the functional interactions of neural and immune signaling systems in pain phenotypes. In pathological states, neural systems interact with the immune system through the actions of a variety of soluble mediators, including cytokines. Cytokines are recognized as important mediators of inflammatory and neuropathic pain, supporting system sensitization and the development of a persistent pathologic pain. Cytokines can induce a facilitation of nociceptive processing at all levels of the neuraxis including supraspinal centers where nociceptive input evokes an affective component of the pain state. We review here several key proinflammatory and anti-inflammatory cytokines/chemokines and explore their underlying actions at four levels of neuronal organization: (1) peripheral nociceptor termini; (2) dorsal root ganglia; (3) spinal cord; and (4) supraspinal areas. Thus, current thinking suggests that cytokines by this action throughout the neuraxis play key roles in the induction of pain and the maintenance of the facilitated states of pain behavior generated by tissue injury/inflammation and nerve injury.

## Introduction

High-intensity mechanical or thermal stimuli will selectively increase the activity of populations of primary afferents, referred to as nociceptors, with the frequency of discharge reflecting the intensity of the stimulus. This input drives activation of second-order neurons, many of which project to the brain. The consequence of this input is to drive a pain state, which at its simplest results in a protective response (e.g., withdrawal of the affected limb) mediated by spinal and supraspinal organization (e.g., nociception) and then at higher-order levels of processing drives a state of negative affect (e.g., pain/suffering) (1). Of note, it is appreciated that in the face of persistent afferent input, as after tissue or nerve injury, there is an increased activation of the afferent and the second-order spinofugal neuron, which drives an enhanced pain response. Such “hyperalgesic” states variously reflect increased responsiveness of the primary afferent and/or the second-order projection neurons, leading to the enhanced pain report. The biology of systems underlying this change in input–output functionality of the spinal dorsal horn has been the subject of considerable interest. One underlying element of this facilitated processing reflects the role played by cytokine signaling in system function.

Classically, three considerations have characterized the actions of cytokines.

1. They are peptides released from immunocompetent cells, notably T cells and monocyte family members (2).
2. This increased production and release is driven by pathological conditions such as tissue injury and infection, which initiate activation of these inflammatory cells.
3. Their perceived role is largely to engage immune signaling and pathologic targets, serving as entities involved in autocrine, paracrine, and endocrine signaling.

Advances in our understanding of cytokine biology have considerably expanded this original profile. It is now clear that aside from immune cells (resident and recruited macrophages, lymphocytes, and mast cells present throughout the neuraxis), cytokines are released from peripheral afferents (Schwann cells and peripheral termini of sensory fibers), as well as from cells within the dorsal root ganglia (DRG) and spinal cord (3). As will be reviewed, this release can indeed be initiated by injury or inflammation, and also by neuronal activity otherwise driven by these injury conditions (4). Cytokine signaling is now known to exert direct effects upon neural signaling through eponymous receptors located on neurons, microglia, and astrocytes, in the spinal parenchyma and in the DRG and brain. Further, while neuronal activation might be the result of receptor-mediated and direct cell-to-cell contact-dependent mechanisms (e.g., gap junction contacts in DRG neurons and satellite cells) (5), soluble extracellular molecules serve to create broader gradients of paracrine- and autocrine-like regulatory networks. These cytokines thus comprise a communication network between immune and neuronal cells. In the context of high-frequency afferent traffic generated by tissue injury, a wave of inflammatory cytokines acts on the terminals of sensory nerve fibers (nociceptors), triggering activation of corresponding pain pathways while neuronal activation leads to a reciprocal activation of a variety of cytokine-generating cells (3). Of note, prolonged inflammation alters nociceptive processing in such a fashion as to yield a persistent pain phenotype even after the inflammation and wounding has resolved, creating a “neuropathic”-like phenotype (6).

A further intriguing complexity is that several of these cytokines, as will be reviewed below, act through signaling to suppress excitatory signaling (e.g., they have an anti-inflammatory phenotype). Finally, current work raises the likelihood that signaling secondary to sustained cytokine and chemokine release and the recruitment of migratory effector cells into the DRG and spinal cord can initiate a feedback loop that results in neuronal injury and subsequently chronic pain. Thus, the balance between repair and proinflammatory factors may determine the rate of progression and outcome of a neurodegenerative process.

Cytokines thus play important roles at the systems level in regulating the functionality of neuraxial systems regulating neurodevelopment, neuroinflammation, and synaptic transmission. Here, we seek to provide an overview focused on a curated list of cytokines identified in the context of neuronal modulation and damage, to play a role in changes in pain processing after tissue and nerve injury, and discuss roles that cytokines play at the interface of the neuronal and immune system interfaces divided across four levels of neuronal organization: (1) peripheral termini; (2) DRG; (3) spinal cords; and (4) supraspinal areas.

## Cytokine Families

Cytokines, from the combination of two Greek words *cyto* (cell) and *kinos* (movement), are defined as a family of low-molecular-weight bioactive proteins or glycoproteins secreted by immune cells and non-neuronal cells (e.g., epithelial cells, fibroblasts, and Schwann cells). Interferon was the first cytokine discovered more than 60 years ago (7). In the absence of a unified classification, cytokines are classified by numeric order of discovery, by kinetic or functional role in inflammatory/immune responses, by primary cell of origin, or by structural homologies shared with related molecules (8). According to structural homologies, cytokines can by classified into groups: tumor necrosis factors (TNFs), interleukins (ILs), interferons (IFNs), colony-stimulating factors, transforming growth factors (TGFs), and *chemo*attractant cyto*kines*, also called chemokines.

Chemokines are small proteins that direct the movement of circulating leukocytes and immune cells. They constitute a family of more than 50 structurally homologous proteins classified in four families according to the location of N-terminal cysteine residues (i.e., CXC, CC, CX3C, or XC). Chemokines affect cells by activating surface receptors that are seven-transmembrane domain G-protein-coupled receptors (GPCRs) and have been implicated in a wide range of inflammatory diseases, such as multiple sclerosis and atherosclerosis (9). These ligands and their respective receptors participate in neuronal and microglial crosstalk (10, 11). The temporal expression of chemokines and their receptors may directly or indirectly contribute to the development of acute pain and the maintenance of chronic pain states.

Historically, cytokines were simply classified according to the functional T-helper (Th) cell group (Th1 or Th2) that produced them. However, recent studies show that cytokines and chemokine display anti-inflammatory and proinflammatory properties producing inhibitory and stimulatory effects in the immune system. As shown in Table 1, properties of a cytokine are dependent on the microenvironment, and most have dual effects according to their context (38, 112, 113). For example, IL-1β is considered a proinflammatory cytokine and can increase neuronal sensitization (17, 18), but it can also regulate inhibitory neurotransmission (15, 16). IL-10 is typically considered to be an immunosuppressive cytokine, which attenuates proinflammatory cytokine release and can reduce antigen presentation. However, IL-10 can also support the activation and proliferation of B cells (39), which can sustain autoimmune attacks. One of the most complex cytokines is TGF-β, which under certain conditions is involved in the differentiation of regulatory T cells (Treg) or in conjunction with IL-6 can drive the differentiation of proinflammatory T cells that produce IL-17 (Th17) (38). Hence, cytokines are characterized by (1) pleiotropy (i.e., a specific cytokine can affect several types of cells), (2) redundancy (i.e., overlapping functions), and (3) cascading signal activation (i.e., one cytokine stimulates the production of additional cytokines) (113, 114).

**Table 1:** Dual effects of cytokines involved in chronic pain^*^.

**Cytokines**	**Major source**	**Receptors**	**Antinociceptive properties**	**Pronociceptive properties**	**Diseases**	**Biologic DMARD (year approved)**
**INTERLEUKINS**						
IL-1β	Macrophages, mast cells, Schwann cells, microglia, astrocytes (12)	IL-1R1 IL-1R2 IL-1Ra	At physiological level, acts as a neuromodulator of LTP (13), assists host defense against infection (14), and can regulate inhibitory neurotransmission (15, 16)	↑Neuronal sensitization (17, 18), ↑mechanosensitivity of C fibers (19), ↑TRPV1 receptor expression in DRG neurons (20), ↑release of proinflammatory cytokines (14)	RA, OA, neuropathic pain, IBD, MS, AD, atherosclerosis (14, 21)	Anakinra (2001) Rilonacept (2008) Canakinumab (2009)
IL-4	Activated T cells (22)	IL-4R1 IL-4R2	↑T cell proliferation, activation of B cells, macrophages, inflammation, and wound repair (22)	Promote the differentiation of monocytes into DCs that support Th1 cell response (23), exacerbate a Th1-dependent model of colitis (24)	Atopic dermatitis Asthma, chronic itch, AD, MS (25–28)	Benralizumab (2017) Dupilumab (2017)
IL-5	Eosinophils, T_H_2 cells, mast cells, NK cells (29)	IL-5R	None	Promote allergic response via ↑eosinopoiesis (29)	Asthma, headache (30, 31)	Mepolizumab (2015) Reslizumab (2016)
IL-6	Monocytes, macrophages (32)	IL-6R sIL-6R gp130	Regenerative processes (classical signaling via IL-6R) (33)	Recruitment of mononuclear cells, inhibition of T cells apoptosis, and Treg cell differentiation (trans-signaling via sIL-6R) (33), ↑TRPV1 in DRG (34), sensitization of nociceptive C-fibers (35)	Arthritis, cancer pain (33, 34, 36, 37)	Tocilizumab (2010) Siltuximab (2014) Sarilumab (2017)
IL-10	Macrophages, DCs, B cells, mast cells, T cells (38)	IL-10R1 IL-10R2	Immunosuppressive activity↓ of proinflammatory release,↓ antigen presentation, ↑release of anti-inflammatory cytokines (39),↑spinal microglial expression of β-endorphin (40) IL-10-deficient mice developed mechanical allodynia (41)	↑Activation and proliferation of immune cells (39), ↑IFN-γ production (42), ↑MHCII expression on B cells, inhibition of the suppression of B cells (38)	RA, MS, SLE, psoriasis, IBS, IBD, post-operative pain, pelvic pain, neuropathic pain (40, 43)	None
IL-13	Th2 cells, CD8+ T cells, mast cells, eosinophils, basophils (44)	IL-13Rα1	Inhibition of the release of proinflammatory cytokines and prostaglandins (45), modulation of pain-facilitating macrophages (46)	Drive skin inflammation (26), potent growth and differentiation factor for B cells (47)	Asthma, breast cancer, chronic itch, RA (26, 45, 48)	Dupilumab (2017) Lebrikizumab (2017)
IL-17	T cells (T_h_17), fibroblasts (49)	Il17RA	Anti-inflammatory effect in the development of experimental autoimmune uveitis (50), maintenance of the epithelial tight junction barrier in the intestinal epithelium during inflammation (51), protection against bacterial-inflammation-induced bone loss (51)	↑Transcription of proinflammatory cytokines (49), direct activation of nociceptors (52), induced hyperalgesia by a TND-dependent neutrophil infiltration (53, 54)	Psoriasis, arthritis (55–57)	Ustekinumab (2009) Secukinumab (2015) Ixekizumab (2016) Brodalumab (2017)
IL-18	Monocytes, macrophages, microglia, astrocytes (58, 59)	IL-18R	None	↑Allodynia and hyperalgesia after intrathecal injection (60) induces astroglial activation (58) and mediates microglia/astrocyte and microglia/neuron interactions (58, 61)	RA, SLE, psoriasis, IBD, bone cancer, neuropathic pain (58, 59, 61)	None
IL-27	Activated APC (62)	IL-27 Rα/WSX-1 TCCR gp130	Suppression of inflammatory immunity via polarization of Tregs (63), ↓expansion of Th17 and IL-17 levels (63–66), and inhibition of osteoclastogenesis (67)	Trigger IFN-γ production by naïve CD4^+^ T cells (62)	Asthma, cancer, metabolic disorders, arthritis (68)	None
IL-33	Macrophage, mast cell, astrocyte, microglia, oligodendrocyte (69)	ST2 (IL1RL1) IL-1RAcP	Single intrathecal treatment with sST2 reduces ongoing CCI-induced hyperalgesia (70)	Oligodendrocytes release IL-33 that activates both astrocytes and microglia to further produce TNF-α and IL-1β (70) and contribute to spinal pain processing (71)	RA, cancer (72–74)	None
IL-35	T_reg_, B cells (75, 76)	IL-35R	Suppression of T-cell proliferation (77) ↓Expression of proinflammatory cytokines, ↓spinal neuronal apoptosis via inhibiting JNK signaling pathways, ↑production of IL-10 (78)	Release of proinflammatory cytokines from mononuclear cells *in vitro* (79)	RA, MS, neuropathic pain (78, 80)	None
**TUMOR NECROSIS FACTOR**						
TNF-α	Macrophages, astrocytes, microglia (81–83)	TNFR1 TNFR2	Nerve demyelination (via TNFR1 signaling) (84)	↑Neuronal sensitization and CGRP release (85–87), stimulation of oligodendrocyte regeneration (via TNFR2 signaling) (84)	RA, cancer, diabetes, IBD (88)	Etanercept (1988) Infliximab (1998) Adalimumab (2002) Certolizumab pegol (2008) Golimumab (2009)
**TRANSFORMING GROWTH FACTOR**						
TGF-β1	Macrophages, Th3 cells (38)	TGF-βR1 TGF-βR2	Development, differentiation, and polarization of Treg (38); inhibition of spinal microgliosis and spinal and astrocyte activation (89)	In association with IL-6, drive the differentiation of Th17 cells to a proinflammatory state (38)	Neurological disorders, arthritis, neuropathic pain, chronic pancreatitis (89–92)	Galunisertib (2019)
**INTERFERON**						
IFN-1α	Macrophages, monocytes, T cells, glial cells, neurons (93)	IFN-α/βR	Analgesic properties: ↓glutamate and substance P release (94)	Potentialization of excitatory synaptic transmission (93)	SLE (95)	None
IFN-γ	CD4^+^ T cells, astrocytes, microglia (38, 96)	IFN-γR	Neuroprotective role and regulation of immunity (97, 98)	Recruitment and activation of microglia (99), ↑excitatory synaptic transmission (94)	Neuropathic pain, lupus, RA, MS, IBD, HLH (99–102)	Emapalumab (2018)
**CHEMOKINES**						
CCL2/MCP-1	Macrophages, monocytes (103)	CCR2	Global suppressive effects on T-cell trafficking and differentiation (38)	Activation of microglia (104), ↑activity of NMDA receptors in dorsal horn neurons (11), recruitment of macrophages (103)	OA, MS, asthma RA, cancer pain, IBD (38, 103)	None
CXCL1/ GRO-α	Macrophages, astrocytes (105)	CXCR2	None	Involve in astroglial–neuronal interaction, central sensitization via NMDA receptors activity (106), attract polymorphonuclear cells toward inflammatory sites (105)	Neuropathic pain (106, 107)	None
CXCL8/IL-8	Macrophages, monocytes, T cells CD8^+^, osteoclasts (108)	CXCR1 CXCR2	Participate in tissue homeostasis (e.g., skin, lung, and joint) via angiogenesis, neutrophil migration, and recruitment (109)	Neutrophil recruitment (109) and angiogenesis (110) in pathological conditions, direct activation of nociceptors in arthralgia (68, 108)	Atherosclerosis, cancer, IBD (109, 111)	None

* *For biologic treatment agents, the date in parentheses represents the initial U.S. approval according to the Food and Drug Administration (FDA). AD, Alzheimer's disease; APC, antigen-presenting cells; DCs, dendritic cells; DRG, dorsal root ganglia; GRO, growth-related oncogene; HLH, hemophagocytic lymphohistiocytosis; IBD, intestinal bowel disease; IFN, interferon; IL, interleukin; MCP, macrophage inflammatory protein; MS, multiple sclerosis; NK, natural killer; NMDA, N-methyl-d-aspartate; RA, rheumatoid arthritis; OA, osteoarthritis; SLE, systemic lupus erythematosus; TGF, transforming growth factor; TNF, tumor necrosis factor; TRPV1, transient receptor potential cation channel subfamily V type 1*.

Physiologically, cytokines are involved in multiple biological functions such as cell differentiation, survival, growth, and metabolism (115). Although broad characterizations of cytokine behavior were aligned with adaptive immune functions, cytokine responses of the innate immune system are important to prevent damage during and following autoimmune attack, inflammation, and infections. In pathological conditions, the imbalance of cytokines participates in the development of the disease and progression leading to damage (114). In the context of the nervous system, some cytokines are considered to function as pain mediators as well as messengers of the immune system. This level of pleiotropy underscores the elegant role these molecules play in communication between the immune system and the nervous system.

## Signaling Pathways

Although the receptors for individual cytokines display specificity for their respective ligands, the subsequent signaling pathways often converge, resulting in nuclear translocation of transcription factors and a secondary transcription of additional downstream mediators. Common signaling pathways activated following cytokine receptor ligation and activation include (1) nuclear factor-κB (NF-κB), (2) the mitogen-activated protein kinases (MAPKs), (3) the Janus kinase (JAK) and signal transducer and activator of transcription (STAT), and (4) the Smad family signaling pathways (114, 116).

### NF-κB Signaling

The most widely studied signaling cascade associated with cytokine signaling is the NF-κB (NF kappa light chain enhancer of activated B cells) family (117). These are a family of highly conserved transcription factors including NF-κB2 p52/p100, NF-κB1 p50/p105, c-Rel, RelA/p65, and RelB, which form functional dimers. Receptors that can activate this cascade include IL-1R and the TNF receptors. In the cytoplasm, NF-κB family members are bound to IκB. In the classical or canonical pathways, proinflammatory cytokine receptors activate an IκB kinase complex (IKKβ, IKKα, and NEMO), which phosphorylates IκB proteins, leading to IκB degradation and the release and translocation of the NF-κB/Rel complexes to the nucleus. In the nucleus, these transcription factors can induce gene transcription alone or in combination with other transcription factors including AP-1 and STATs (118).

Some of the target genes include other proinflammatory cytokines, like IL-6 and IL-8. In some instances, there is an alternative pathway through signaling of cytokine receptors like the lymphotoxin-β receptor (LTβR and TNFRSF3) (119). These activate Nck-interacting kinase [NIK; MAPK kinase kinase (MAPKKK) 14], which in turn activates IKKα complexes that phosphorylate NF-κB2 p100. Phosphorylation of NF-κB2 p100 then leads to its ubiquitination and proteasomal processing to NF-κB2 p52/RelB complexes that translocate to the nucleus and induce target gene expression (117).

### MAPK Signaling

The MAPKs are generally divided into the p38 stress-activated protein kinase (SAPK)/Jun amino-terminal kinase (JNK) and extracellular signal-regulated kinase (ERK) pathways (120). These kinase pathways are activated by a variety of environmental stresses, growth factors, GPCR agonists, and inflammatory cytokines. In the MAPK cascades, there are tiered activation steps. The membrane proximal MAPKKK kinases (MAPKKKKs) or GTPases activate MAPKKK, which mediate phosphorylation and activation of MAPK kinases (MAPKKs), which in turn phosphorylate and activate MAPK. p38 MAPK is activated by MKK3/MKK6 and is involved in the regulation of HSP27, MAPKAPK-2 (MK2), MAPKAPK-3 (MK3), and several transcription factors including ATF-2, Stat1, the Max/Myc complex, MEF-2, Elk-1, and indirectly CREB via activation of MSK1 (121).

Stress signals are delivered to the JNK family cascade by small GTPases of the Rho family (Rac, Rho, and cdc42). As with the other MAPKs, the membrane proximal kinase is a MAPKKK, typically MEKK1–MEKK4, or a member of the mixed lineage kinases (MLKs) that phosphorylate and activate MKK4 (SEK) or MKK7 and that phosphorylate the SAPK/JNK kinases, which then translocate to the nucleus where they can regulate the activity of multiple other transcription factors (122).

The ERK signaling cascade is activated by receptors involved in growth and differentiation including receptor tyrosine kinases (RTKs), integrins, and ion channels. The receptors signal through cascades that include small GTP-binding proteins (Ras and Rap1), which in turn activate a MAPKKK (Raf), a MAPKK (MEK1/MEK2), and then Erk MAPK (123). Erk dimers can regulate targets in the cytosol and also translocate to the nucleus where they phosphorylate a variety of transcription factors regulating gene expression related to growth, migration, and differentiation. As an example of signaling complexity for cytokines, TNF acts through two receptors, TNFR1 and TNFR2, which drive MAP kinase activation and enhance inflammatory responses by secondary IL-1, IL-6, and IL-8 release following the transcription of their target genes (124).

Activation of neuronal TNF receptors drives MAPK activation, which enhances inflammatory response by increasing IL-1, IL-6, and IL-8 release. IL-1 for instance is involved with cyclooxygenase (COX) upregulation within the DRG, inducing neuronal sensitization. Moreover, sensitization of ion channels in neuronal cells is involved with pain processing (125). IL-6 has been shown to induce JAK and protein kinase C (PKC) activation, which enhances the ion channel transient receptor potential (TRP) cation channel subfamily V member (TRPV1) sensitivity. In fact, JAK and PKC inhibitors decrease TRPV1 sensitization (126, 127). However, not only does this sensitization apply for the primary afferent, but it also seems that cytokines can induce neuronal sensitization in other anatomical levels such as cells in the DRG, dorsal horn of the spinal cord, and supraspinal areas (128). In fact, peripheral inflammation increases the expression of IL-1β and COX in the DRG, cascades known to be involved with neuronal network sensitization (18). It is thus noteworthy that TNF and IL-1β induced sensitization of cells in the dorsal horn and increased pain hypersensitivity (hyperalgesia) by enhancing α-amino-3-hydroxy-5-methyl-4-isoxazolepropionic acid (AMPA)- or *N*-methyl-d-aspartate (NMDA)-induced currents. Further, IL-1β and IL-6 suppressed typical inhibitory gamma-aminobutyric acid (GABA)- and glycine-induced currents (17). Accordingly, TNF and IL-1β enhanced NMDA receptor phosphorylation in the trigeminal nucleus in mesencephalic (trigeminal nucleus) and also increased NMDA current in the hippocampus (129).

### Smad Signaling

The Smad family of transcription factors is largely downstream of the TGF-β and bone morphogenetic protein (BMP) superfamilies (130). In general, signaling is initiated with ligand-induced activation of serine/threonine receptor kinases and phosphorylation of the cytoplasmic signaling molecules Smad2/Smad3 for the TGF-β/activin pathway, and Smad1/Smad5/Smad9 for the BMP pathway. Activated Smads regulate diverse biological effects by partnering with other transcription factors, resulting in transcription of specific cell state-associated target genes (131). This family has inherent regulatory negative feedback loops with inhibitory Smads (I-Smads) 6 and 7, which are also induced by both TGF-β and BMP signaling. The TGF-β and BMP pathways are cross-regulated by MAPK signaling. Moreover, in certain contexts, TGF-β signaling can also affect the Erk, SAPK/JNK, and p38 MAPK pathways independent of Smad activation (116).

### JAK/STAT Signaling

Over 50 cytokines and growth factors use the JAK/STAT pathway for signaling. After receptor ligation, the JAK proteins are phosphorylated, and activated JAKs then phosphorylate STAT monomers, leading to dimerization, nuclear translocation, and DNA binding. Although there are four JAKs (JAK1, JAK2, JAK3, and TYK2) and seven STATs (STAT1, STAT2, STAT3, STAT4, STAT5a, STAT5b, and STAT6) in mammals, the number of potential combinations alone does not fully explain the pleiotropy in signaling (22, 34, 132). For example, IL-6R and IL-10R signaling both result in STAT3 activation through JAK activation; however, one cytokine is mostly proinflammatory, and the other is considered anti-inflammatory. There may be differences in STAT use for some cytokines, whereas other cytokines like IL-27 strongly activate more than one STAT protein (STAT1 and STAT3). To add to the complexity, multiple STATs may bind to the same target site due to shared specificities. In addition STAT proteins can be phosphorylated on serine residues to influence their DNA binding pattern, and STAT signaling influences epigenetic changes. Some STAT proteins have extra nuclear functions, for instance, at the mitochondrion level (133, 134).

### Cytokine-Induced Peripheral Transcription

**Figure 1:**
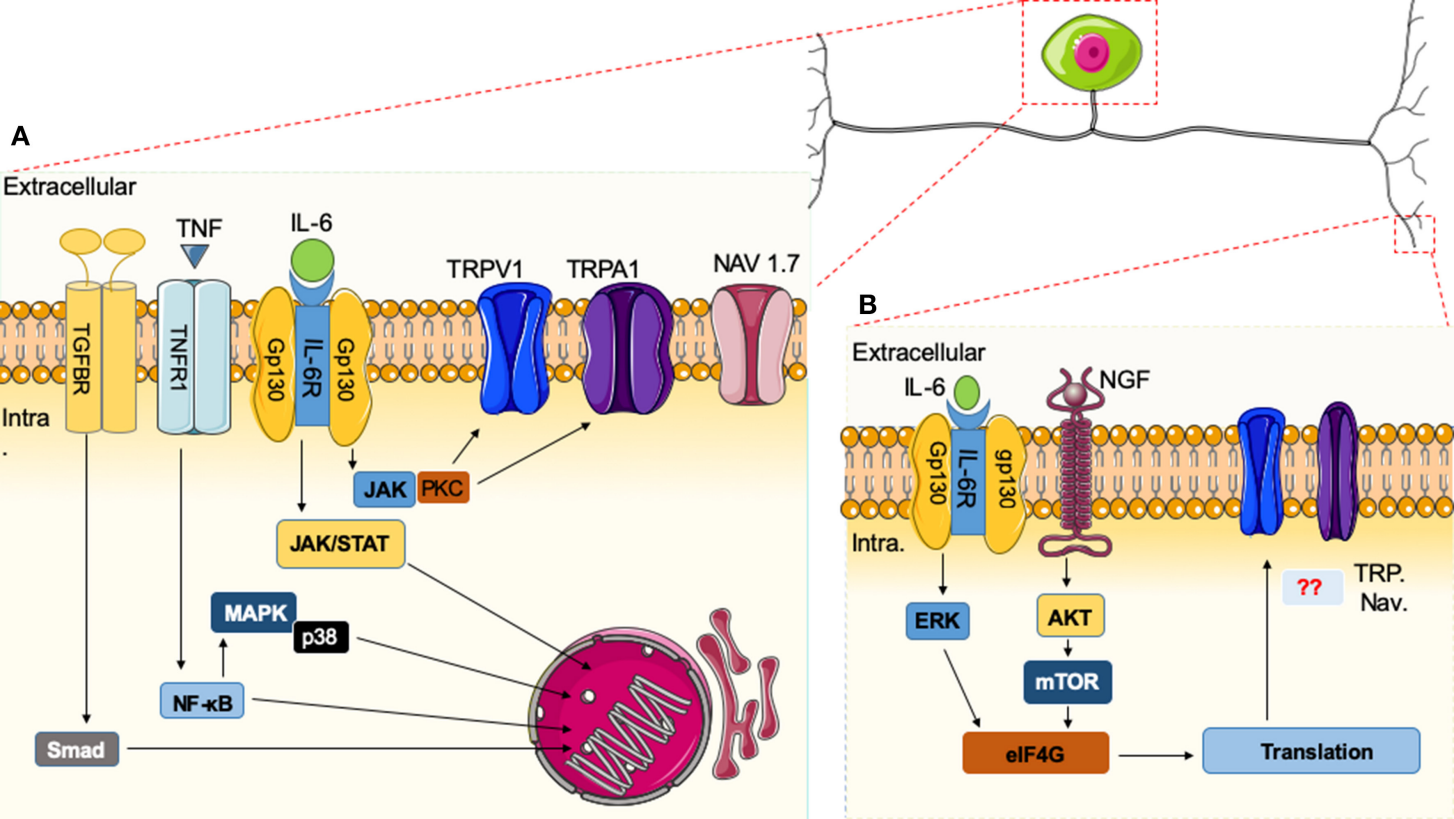
Immune system and nociceptor activation. **(A)** Cytokines released in the vicinity of the cell body of nociceptors can induce specific receptor activation and signaling cascades. Upon activation of nuclear factor (NF)-κB, mitogen-activated protein kinase (MAPK)/Janus kinase (JAK), and Smad transcription factors present in the cytoplasm are phosphorylated and translocated to the nucleus, leading to the expression of target genes, resulting in biological responses. Alternatively, downstream modulators like protein kinase C (PKC) can sensitize neurons through effects on ion channels (e.g., TRPV1 or Na^+^ channels). **(B)** In the peripheral terminus, there are additional cytokines that signal through the extracellular signal-regulated kinase (ERK) pathway and the AKT/mTOR pathways leading to phosphorylation of eIF4E, which can regulate local protein synthesis in the peripheral processes of sensory afferents. It remains unknown how these pathways directly intersect with the activities of ion channels.

While it is well-documented that nuclear translocation of transcription factors is initiated by cytokine signaling, it has recently been demonstrated that cytokines also can induce nociceptive plasticity by local protein synthesis in the *peripheral* processes of sensory afferents (135). It is well-established that IL-6 expression is increased in arthritis and peripheral nerve injury. Likewise, nerve growth factor (NGF) levels are elevated in inflammatory and neuropathic pain states (36). IL-6- and NGF-induced mechanical hyperalgesia is reversed by rapamycin (135). Moreover, the IL-6R signals through the ERK pathway and the NGF receptor signals primarily through the AKT/mTOR pathway leading to phosphorylation of the CAP-binding protein eIF4E. Finally, phosphorylation of eIF4E enhances rapid changes in the translational control of gene expression in sensory neurons, and this effect is linked to mechanical allodynia (136). Although cytokines can induce nociceptive plasticity by local protein synthesis in the peripheral processes of sensory afferents, it remains unclear how these pathways directly intersect with the activities of ion channels or GPCR. In Figure 1, we schematically illustrate these pathways and actions in the cell body of a nociceptor (Figure 1A) and the peripheral terminus (Figure 1B).

## Peripheral Nociceptor Terminal

Irritants or immunogens such as carrageenan administered in the hindpaw induce a transient hyperalgesia that is prevented by non-steroidal anti-inflammatory drugs (NSAIDs). However, inflammatory mediators such as prostaglandins into the same site induced prolonged hyperalgesia, described as latent or “primed” state, that is not prevented by NSAIDs (85, 137). In the same manner, during chronic pathophysiological conditions such as arthritis, cytokines induce neuronal sensitization and priming of the primary afferent (85), of the DRG (18), and as reviewed below at supraspinal levels (128). Indeed, in an arthritic murine model [K/BxN passive serum transfer (138)], the mouse shows an early phase characterized by inflammation, increased TNF production, and pain and a late phase characterized by the inflammation's resolution, decreased TNF, but persistent pain (41). These data suggest that cytokines play a role in priming peripheral nociceptors.

Peripheral termini of nociceptors form arborization-like structures in the skin, muscles, bone, joints, and viscera (139). These locations, in proximity to many different cells (e.g., keratinocytes and immune cells), facilitate the immune modulation of nociceptor function. Among the many different inflammatory diseases, arthritic diseases like rheumatoid arthritis (RA) have been studied for the pathophysiology of localized peripheral inflammation and pain. Notably, arthritic diseases have been described to involve an imbalance of cytokines (114, 140). In RA, studies highlight an early alteration of cytokine and chemokine levels months to years before the onset of joint swelling, particularly in patients with arthralgia (i.e., joint pain without observable clinical signs of disease such as joint redness or swelling) (48, 141, 142). Serum cytokine profiles also differ in RA patients with specific autoantibodies. Anti-citrullinated peptide autoantibodies (ACPAs) are present in a major subset of RA patients (around 60–70%) (143), are serological markers for the diagnosis of RA (144), and are prognostic factors for more aggressive joint diseases (145). In the sera of RA patients with ACPAs, there was an increase in the IL-5, IFN-1α, TNF, and IL-13 levels. In contrast, there were specific increases in eotaxin and RANTES (i.e., regulated on activation normal T cell expressed and secreted) levels in the sera of RA patients who did not have any detectable ACPAs (48). In animal models of arthritis, including antigen-induced arthritis (AIA), collagen antibody-induced arthritis (CAIA), and collagen-induced arthritis (CIA), IL-6 has been implicated as a key factor in peripheral pain mechanisms (36). Direct IL-6 or IL-6/sIL-6R administration into knee joints in rodents induced a long-lasting sensitization of nociceptive C-fibers, contributing to mechanical hypersensitivity (35).

In models of inflammatory pain, such as carrageenan administration in the hindpaw, infiltration of macrophages and the local release of TNF play a key role in the development and sensitization of peripheral afferents (85). Activation of neuronal TNF receptors increases the production of IL-1β, IL-6, and CCL2 [formerly known as monocyte chemoattractant protein-1 (MCP-1)]. Both IL-1β and IL-6 have been shown to have activity in acute inflammatory and chronic pain (146–148). Indeed IL-1β enhanced pain transduction and conduction via modulation of ion channels such as TRP ankyrin 1 (TRPA1), TRPV1, and Nav1.7. CCL2 also contributes to macrophage recruitment (104, 149). Thus, these factors serve to perpetuate a feedback loop between neuronal sensitization and cytokine production during tissue injury or inflammation.

Other studies have suggested that additional cytokines play key roles in the induction of inflammatory pain in models of arthritis, including IL-8, IL-17, and IL-27 (55, 58, 68). The chemokine IL-8 and its receptor CXCR2 are involved in sensitization of afferent nociceptors in ACPA-induced arthralgia. Interestingly, recent studies demonstrated that a single injection of human IgG ACPA or monoclonal murinized IgG ACPA antibodies isolated from RA patients is capable of initiating pain without paw swelling when injected into rodents (68). This effect of ACPAs is associated with osteoclast activation, at least *in vitro*, which via the release of the chemokine CXCL1 (an analog to human IL-8) mediates their pronociceptive effects (68, 108). IL-8 has also been implicated in conditions such as chronic low back pain (LBP). Krock et al. showed a specific upregulation of this chemokine in the cerebrospinal fluid of LBP patients with degenerating disks and a reduction of disk degeneration and chronic back pain in a mouse model (148). In a neuropathic orofacial pain condition, the burning mouth syndrome, patients present an elevated level of plasma IL-8, and this signature directly correlates with pain and depressive symptomatology (150). Hence, IL-8 has been implicated in the periphery for several different pain phenotypes.

As described above, IL-6 has pleiotropic roles associated with pain and inflammation. Another cytokine that also shares the gp130 common receptor chain and signals through the JAK/STAT pathway is IL-27. IL-27 is a heterodimer formed by the Epstein–Barr virus-induced gene 3 (EBI3) and IL-27 p28 subunits, which binds to a receptor composed of the gp130 common receptor chain and IL-27Rα (i.e., WSX-1 or TCCR) (62). Sasaguri et al. showed that IL-27 signaling constitutively contributes to control of thermal (heat and cold) and mechanical sensitivity (151). In an arthritis model, IL-27 attenuates disease development and histological disease severity (i.e., cell infiltration in the joint, synovial hyperplasia, and joint erosion) by reducing the expansion of Th17 cells and IL-17 levels (63–66), which can reduce nociception (Table 1). In osteoimmunology, IL-27 plays a critical role in limiting bone erosion by inhibiting osteoclastogenesis (67). Osteoclast activity is directly involved in pain development and reduced with the use of osteoclast inhibitors such as bisphosphonates or denosumab (152). The suggested mechanisms of this action include mechanical stabilization in bone pain from trauma and also changes in pH and acidosis in bone pain from cancer. Nociception could be promoted by acidosis in which H^+^ protons can directly activate specific ionic or receptors sensitive to protons such as TRPV1 and the acid-sensing ion channel (ASIC) family (153, 154).

## Dorsal Root Ganglia

It has become apparent that the excitability of the afferent input circuitry reflects the functional complexity of the DRG system. DRG neurons are supplied by a fenestrated vasculature that lies outside the blood–brain barrier (BBB), slowing the passage of molecules that are normally excluded from the neuraxis (155). This exposure of the afferent cell bodies to circulating products, including cytokines, may partly explain why circulating neurotoxic agents (e.g., chemotherapeutics) preferentially accumulate and injure cells within the DRG, inducing a sensory rather than a motor neuropathy (156). DRG neurons are additionally supported by satellite glial cells (SGCs), which envelop them and display gap junction linkages between these two cell types (157). During inflammation, SGCs display enhanced activation, increased TNF production, and neuronal excitability (73). An increase of gap junctions has been observed in pain-generating conditions, and this correlates with enhanced neuronal excitability (157). Importantly, peripheral inflammation or nerve injury causes DRG neuronal sensitization, leading to a spreading activation of SGCs through gap junctions and to the expression and release of IL-1β from SGCs.

Current work highlights an important role of macrophages in response to inflammation and cytokine signaling. A major source of endogenous cytokine production is from these resident and migratory DRG macrophages. In arthritic conditions (e.g., osteoarthritis and RA), macrophages infiltrate into the DRG and acquire a phenotype resembling that of TNF-stimulated macrophages, suggesting a role of these cells in the maintenance of arthritic pain (158). *In vitro*, macrophages stimulated with TNF promote release of calcitonin gene-related peptide (CGRP) by nociceptors, which is consistent with their pronociceptive effect (159). *In vivo*, TNF is involved with sensitization of nociceptive fibers and elicits a rapid increase of CGRP release from the peripheral termini of nociceptors (86, 87). Other than macrophages, during inflammation, SGCs become activated, increasing TNF production, and enhancing neuronal excitability (73). In neuropathic pain, a key role of TNF in the DRG has been demonstrated by lentivirus-mediated silencing of TNF in DRG, which attenuated the pain phenotype and reduced neuronal cell death in mice with an L5 transection (160).

DRG cells produce additional cytokines such as IL-1β and IL-6 and chemokines such as CCL2 or CXCL1 within the DRGs that are involved in pain signaling (3, 161). Interestingly in osteoarthritis, a high CCL2 production is associated with elevated numbers of macrophages in the DRG and a high level of CGRP in DRG neurons (162, 163). Mice lacking the CCR2 receptor (global knockout) fail to develop mechanical allodynia in nerve injury models (164). Accordingly, we suggest that during inflammation, a neuro-crosstalk can occur in the DRG where inflammation triggers cytokine and chemokine release from local/infiltrating macrophages and neurons, contributing to development and maintenance of facilitated states of excitability in the local DRG circuit. Such enhanced excitability would contribute to an enhanced afferent input into dorsal horn second-order neurons.

Several cytokines, IL-6 for example, can excite DRG neurons directly by rapid effects that do not require gene transcription but are likely to involve phosphorylation of different ion channels, such as the TRP family. IL-6 is a pleiotropic cytokine with a pivotal role in the pathophysiology of arthritis and pain sensitization through increasing neuronal calcium mobilization, action potential generation, and ion channel sensitization. IL-6 acting through IL-6R and gp130 drives JAK and PKC activation, which enhances TRPV1, inducing excitability of nociceptive TRPV1^+^ DRG neurons (34, 124). Correspondingly JAK and PKC inhibitors decrease TRPV1 sensitization (34).

Gap junctions in the DRG can provide direct communications between neuronal cell bodies and SGCs. An increase in gap junctions has been observed in pain condition and seems to enhance neuronal excitability and thus elicit pain (157). Importantly, peripheral inflammation or nerve injury causes sensitization of neurons, innervating peripheral tissues, and spreading of activation of SGCs through gap junctions, which leads to the expression and release of IL-1β from SGCs. IL-1β has been shown to increase TRPV1 expression in DRG neurons. Moreover, IL-1RI antagonism reduces thermal hyperalgesia antigen-induced arthritis (20). IL-1β has been shown to act in a p38 MAPK-dependent manner, to increase the excitability of nociceptors. Indeed, IL-1β relieves resting slow inactivation of tetrodotoxin-resistant voltage-gated sodium channels and also enhances persistent TTX-resistant current near the threshold (165). These IL-1β actions on nociceptors have facilitatory effects in neurotransmission, which at least in part explains the hyperalgesic effect seen with the direct application of IL-1β or the endogenous production and release of IL-1β within the DRG.

Beyond the effect of IL-1β on ion channel sensitization, it has been shown that intraplantar IL-1β can induce persistent hyperalgesia, which is dependent on GPCR kinase 2 (GRK2) and IL-10 downregulation. GRK2 plays a regulatory role in the inflammatory response as studied in arthritis models. Reduction of GRK2 in peripheral macrophages markedly prolonged hyperalgesia and pain behavior in response to an intraplantar injection of IL-1β or the inflammatory agent carrageenan (166). The reduction of GRK2 in macrophages is associated with the transition from acute to persistent hyperalgesia due to the lack of IL-10 production. Moreover, local anti-IL-10 treatment in the paw did not influence IL-1β-induced hyperalgesia, indicating that IL-10 signaling in the spinal cord or DRG is required for spontaneous resolution of hyperalgesia. Corroborating these data, our group recently showed that mice deficient in IL-10 rapidly developed mechanical allodynia that did not recover, suggesting that this cytokine also plays a key role in the acute and chronic phases of pain-like behavior (41).

These data suggest that beyond changes in the peripheral terminus nociceptor, the DRG plays an important role in the development of pain states. Thus, cytokines produced by local cells or released into the DRG are involved with the facilitation of nociceptive stimulus, inducing subsequent dorsal horn spinal activity.

## Spinal Cord

The peripheral nociceptor forms an excitatory synapse with second-order neurons in the dorsal horn of the spinal cord to initiate transmission in the central nervous system (CNS). This synapse can be modulated by local interneurons, microglia, and astrocytes. These last cells constitute a component serving to alter the input–output function of the dorsal horn. Inflammatory cytokines maintain enhanced pain signaling through modulating the central terminals of nociceptors and/or spinal cord neurons. In naïve animals, intrathecal delivery of cytokines can induce a direct pronociceptive effect, leading to mechanical, and/or thermal hyperalgesia, which has been observed after intrathecal injection of IL-1β (15, 16, 167, 168), IL-18 (60), TNF (15, 16, 167), IL-6 (15), CXCL1 (107), CX3CL1/fractalkine (169), or CCL2/MCP-1 (170) among others. Conversely, a pronociceptive phenotype is reduced in mice lacking such cytokine or chemokine signaling or after the administration of anti-cytokine antibodies or cytokine receptor antagonists. In pathologic conditions, chronic intrathecal administration of IL-1 receptor antagonist (IL-1ra) prevents pain induced by nerve injury in mice, and a single intrathecal injection of IL-1ra induced a long-lasting attenuation of mechanical hypersensitivity in the same model (171). Intrathecally, coadministration of IFN-β and anti-TNF antibodies permanently reversed mechanical allodynia in males in the murine K/BxN serum transfer model of RA (41). Moreover, intrathecal delivery of cytokines such as TGF-β or IL-13 can also induce an antinociceptive effect in neuropathic pain condition (46, 89, 158). TGF-β can inhibit excitatory synaptic transmission in the spinal cord (172) and neuropathic pain along with glial activation and neuroinflammation in the spinal cord (89, 172).

Considering that cytokines play a role in the development and maintenance of pain at the spinal cord level, we raise the question of where these neuraxial cytokines are produced and where they act. Microglia serve as spinal-resident macrophage-like cells and display a rapid response to increased afferent traffic and to pathological changes in the CNS. Spinal microglial activation has been demonstrated in several pain conditions [for review, see (173), including mononeuropathies after peripheral nerve injury (174), polyneuropathies after chemotherapy (175, 176), or in diabetic models (177), chronic inflammatory pain (178), and cancer pain (179), although some data are conflicting (180)]. Microglia participate notably in the regulation of neuroinflammation that contributes to the pathogenesis of pain (173). Signals that activate microglia converge on intracellular signaling cascades frequently involving the phosphorylation of p38 MAPK, triggering production and release of TNF, IL-1β, and IL-18; increased expression of COX; and subsequent synthesis of prostaglandin E2 (PGE_2_) (18, 181, 182). These neuromodulators then lead to enhanced dorsal horn excitability, which ultimately serves to enhance receptive fields, increase output of the pain-relevant sensory message to supraspinal areas, and enhance pain behavior.

Astrocytes have a key role in neurotransmitter recycling, regulation of blood flow, energy metabolism, synaptogenesis, and synaptic transmission (183–187). Astrocytes signal physically through gap junctions (e.g., connexin Cx43), facilitating intercellular transmission (188, 189). Moreover, it has been demonstrated that upregulation of Cx43 triggers release of chemokines, ATP, and glutamate, which ultimately induces nociceptor sensitization (185, 186). Astrocytes have been shown to play both beneficial and detrimental roles, depending on the nature of the injury or disease, that differ in their functions (190). Thus, spinal cord astrocytes can generate IFN-α, which have an antinociceptive effect mediated through the mu opioid receptor (94). In addition, recent data from our group have shown that the post-inflammatory allodynia from an arthritis model may be robustly regulated by downstream effectors activated through IFN-β and interferon-inducible factors, including IL-10 and IL-1ra (41). In this model, the allodynia only subsided when anti-TNF therapy was combined with supplemental IFN-β, indicating that chronic pain treatment might require modulation of multiple pathways (41).

Astrocytes can also modulate pain through IL-33 production. IL-33 is a member of the IL-1 superfamily but is active after transcription and is deactivated by caspase cleavage. It binds to the ST2 (Il1rl1) receptor, encoded by the *IL1RL1* gene, and the coreceptor IL-1 receptor accessory protein (IL-1RAcP). After receptor engagement, the MyD88 signal cascade is activated similarly as after IL-1R and IL-18R activation. This cytokine has a pronociceptive effect with intrathecal injection (191, 192). In addition, inhibiting the IL-33/ST2 pathway reduced nociceptive behavior in murine models of pain, including cancer and chemically induced inflammation (72, 192, 193).

The effects of the intrathecal injection of cytokines cited above directly support the key roles played by spinal cord cytokines in pain states. As these cytokines are not acting independently, it is not surprising that these agents display important interactions in different pain states reflecting their facilitatory and suppressive interactions. As an example, intrathecal administration of recombinant IL-27 induced antinociception dependent on IL-10 during the maintenance phase of peripheral neuropathy (194). Also, intrathecal delivery of IL-35 in an experimental murine model of autoimmune encephalomyelitis (EAE) reduced pain behaviors (i.e., facial allodynia and grimacing), which was noted to occur through an upregulation of an inflammatory cytokine, IL-10 (195). IL-35 has been very recently highlighted as a candidate target for diabetic neuropathic pain (DNP) treatment (78). In fact, in a streptozotocin-induced DNP rat model, other than a protective effect against inflammatory response, IL-35 injected intrathecally reduced allodynia via inhibition of JNK signaling (78).

Second-order dorsal horn neurons express cytokine receptors such as TNFR1, TNFR2, IL-1R, and IL-6R. Cytokine released at the spinal cord level by resident cells (e.g., microglia/astrocytes) can induce sensitization of the secondary neuron, leading to supraspinal areas of activation where pain is processed and perceived as an uncomfortable sensation (139). In fact, IL-6, TNF, and IL-β enhance spontaneous post-synaptic current (sEPSCs) in the spinal cord by both increasing excitatory synaptic neurotransmission and suppressing inhibitory synaptic transmissions (160). Taken together, these studies show that therapeutically targeting peripheral inflammation will not necessarily affect persistent pain. However, modulating multiple pathways at the spinal level might be an effective way to prevent the development of chronic pain and to alleviate ongoing pain.

## Supraspinal Areas

Changes in higher-order functions such as anxiety or depression are critical components of pain phenotypes, especially in the context of a chronic pain state (196, 197). Studies in animal models (99, 198, 199) and *in vivo* positron emission tomography (PET) associated with magnetic resonance imaging (MRI) in humans (200, 201) are consistent with the fact that chronic pain states lead to an alteration of glial function in the brain. Current thinking emphasizes the critical role that cytokines can play in regulating depressive states, through their effects upon key aminergic pharmacological systems regulating depressive states such as increased monoamine transporter activity (202), reduced cofactor availability (203), and reduced expression of glutamate transporters and increased glutamate release from astrocytes (204).

Cytokines play key roles in supraspinal modulation of pain transduction. Consistent with this assertion are the findings that intracerebroventricular (ICV) injection of TNF, IL-1β, and IL-6 induced hyperalgesia (205–207) and that blocking these cytokines in the brain reduced the behaviorally defined expression of a pain state (208). Of note, mechanistically, it is not clear whether this pain state reflects the role of an enhanced supraspinal response to the ascending pain stimulus or an activation of descending facilitatory/loss of descending inhibitory system (209).

Microglial activation (Iba1 marker) after nerve injury is observed in specific brain regions involved in pain and affect. These regions include not only the thalamus and somatosensory cortex but also limbic regions considered to be affiliated with the affective component of the pain response (210). Importantly, as in the spinal cord, glial cells are thought to be a major source of cytokines and chemokines in the brain (204), and the activation of these cells is considered to play a key role in anxiety and depression, comorbidities associated with chronic pain states (211, 212). Other works support the involvement of microglial activation in prefrontal cortex, amygdala, and hippocampal neurons associated with an overproduction of TNF in neuropathic pain and chronic-pain-associated depression (211, 213). Moreover, upregulation of IL-10 and IL-1β is found in the contralateral–ventrolateral orbital cortex (VLO) of rats with spared nerve injury (SNI), and IL-1β expression and glutamatergic neurotransmission are enhanced in the prefrontal cortex (PFC) of mice with neuropathic pain (214).

Cytokine activation in the brain on the affective-motivation component of pain processing is an exciting component of the role played by cytokines in pain processing. Considerable work has demonstrated that circulating inflammatory markers (e.g., IL-1β, TNF, IL-6, and C-reactive protein) are important covariates for depression and anxiety in humans (215, 216).

## Cytokines as Therapeutic Targets

Although not the focus of this review, it would seem remiss not to briefly comment on some of the advances seen with the advent of approved biologic therapies. As described previously, cytokines and chemokines have a key role in disease-associated pain and therapies exerting an action on cytokine release or activity, which is really effective [Table 1; (182, 183)]. Accumulating data suggest that in a variety of pain states, there is a strong covariance between circulating proinflammatory cytokine messages and the pain states in fibromyalgia (217) or painful (vs. non-painful) neuropathies (218, 219). Of note, TNF antagonism improved depressive symptoms in patients with high baseline inflammatory biomarkers (220). While these endpoints do not directly impact upon pain signaling, they provide an important covariate between chronic pain and comorbidities that can enhance the chronic pain states (221).

The bulk of clinical data describe the relief of pain in the treatment of inflammatory states like RA using agents that block the activity of key cytokines like IL-6 and TNF. Conventional synthetic disease-modifying antirheumatic drugs (csDMARDs) such as methotrexate or sulfasalazine can attenuate cytokine release (222–226), but the biologic disease-modifying antirheumatic drugs (bDMARDs), which have a direct effect on the function and levels of circulating cytokines, have been shown to be more effective alone or in combination with csDMARDs [Table 1; (124, 227–229)]. The success of these agents is reflected in the development of agents with similar targets. Notable groups of agents include anti-TNF (including infliximab, adalimumab, certolizumab pegol, etanercept, or golimumab) and anti-IL-6 or IL-6 receptor antagonists (i.e., tocilizumab, sarilumab, clazakizumab, and olokizumab) (115). However, remission of inflammation by clinical parameters has not universally been associated with complete relief of pain, and residual pain with neuropathic features can persist (178). More recently, agents that intercede with signaling, notably the JAK inhibitors, may add to the armamentarium of agents that can reduce pain as well as inflammation (230, 231). The development of therapeutic agents that target individual cytokines and their signaling pathways is a promising and exciting area that has been extensively reviewed by others (232, 233). These therapies hold significant promise for the future, and further investigations into their level of anatomic activity will hopefully yield insights into individualized therapeutic plans.

## Conclusion

**Figure 2:**
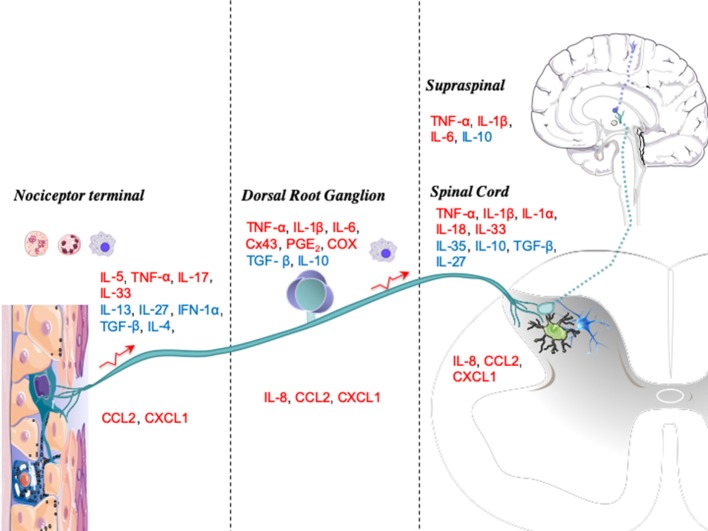
Anatomic levels of cytokine interactions with nociceptive processing. Upon injury or infection, mediators such as immune cytokines are released locally by resident or blood-derived immune cells. The peripheral terminals of nociceptors, dorsal root ganglia (DRGs), and spinal cords have several receptors for these mediators, which activate signaling cascades that modulate nociceptive activity. Cytokines are displayed as pronociceptive (red) or antinociceptive (blue).

The primary emphasis of this review has been on reviewing the role of cytokines at the levels of the peripheral terminus, the DRG, the spinal dorsal horn, and supraspinal circuits (Figure 2). Although pain arises from different conditions, some mechanistic components are conserved across pain states:

- Peripheral nerve fibers (nociceptors) are directly exposed to circulating products like cytokines and detect environmental stimuli (thermal, mechanical, or chemical nature) and stimulate excitation of second-order neurons at the spinal cord.
- In the DRG, the somata of nociceptors are surrounded by SGCs and macrophages. As noted, the DRG lies outside of the conventional BBB restriction, leading it to be directly exposed to these circulating proteins and other danger signals. These peripheral stimuli drive cytokine secretion from SGCs and macrophages, contributing to inflammatory signaling cascades and persistent pain.
- The dorsal horn in the spinal cord receives information from nociceptors.
- The incoming information is processed by complex circuits involving excitatory and inhibitory interneurons and transmitted to projection neurons to several supraspinal areas in the CNS. Spinal cord astrocytes and microglia are described as key cells in the mechanism of pain processing in several pain models (234).

In Figure 2, we graphically summarize key cytokines that play a role in these four major neuraxial components (i.e., the peripheral terminal, DRG, spinal cord, and brain). At each anatomic level, we note the relevance of the several local systems (neuronal, glial, and inflammatory cells) that contribute both as a source of cytokines and as a target for these molecules functioning in an autocrine-/paracrine-like fashion.

This review serves to emphasize the multiple levels at which cytokines may be released and act to alter the nociceptive phenotype and reflect the role of a local paracrine or autocrine function. It is clear, however, that this position does not exclude the likelihood that the circulating cytokine profile observed in a variety of inflammatory and injury states might contribute to the abnormal pain and depression though a circulating delivery. Of note, the presence of these circulating proinflammatory products and the accessibility of these products to neuraxial components such as the peripheral terminal and the DRG point to potential interactions. The ability of glia such as astrocytes and perivascular macrophages to sample circulating products, along with the evident role played by glia in CNS function, points to the likelihood that circulating products can modify function throughout the neuraxis. We therefore conclude that targeting inflammatory cytokine and chemokine signaling may provide additional strategies in the therapeutic intervention of chronic pain. However, we note that despite recent promising advances, any single agent is unlikely to be uniformly effective, and future studies in this area are warranted.

## Author Contributions

All authors contributed to the drafting and revision of the manuscript.

### Conflict of Interest

The authors declare that the research was conducted in the absence of any commercial or financial relationships that could be construed as a potential conflict of interest.
